# Disease Models & Mechanisms helps move heart failure to heart success

**DOI:** 10.1242/dmm.049634

**Published:** 2022-05-20

**Authors:** Kirsty Hooper, Julija Hmeljak

**Affiliations:** The Company of Biologists, Bidder Building, Station Road, Histon, Cambridge CB24 9LF, UK

**Keywords:** Cardiac regeneration, Heart disease, Heart failure

## Abstract

Heart failure affects ∼64 million people worldwide, resulting in high morbidity, mortality and societal cost. Current treatment strategies are primarily geared at slowing the progression to an advanced disease state, but do not reverse or cure heart failure. A more comprehensive understanding of the underlying biology and development of preclinical models of this heterogeneous group of disorders will improve diagnosis and treatment. Here, we summarise recent preclinical and translational research in this area published in Disease Models & Mechanisms. We also discuss how our Journal is propelling this field forward by launching a Special Issue and ongoing subject collection, ‘Moving Heart Failure to Heart Success: Mechanisms, Regeneration & Therapy’.

Heart failure is characterised by weakening or stiffening of this vital organ, resulting in impediment of its function. Heart failure is most often associated with ischaemic heart disease or high blood pressure, but also arises due to congenital heart disease, cardiomyopathy, arrhythmias, heart valve abnormalities and inflammatory disorders. Globally, heart failure affects ∼64 million people (Lippi and Sanchis-Gomar, 2020). Various treatments can support and improve heart function, such as lifestyle changes, therapeutics, medical devices and surgery. However, when patients progress to advanced disease, treatments are limited to heart transplantation and mechanical assist devices, each associated with a high degree of morbidity. Despite advances in diagnosis and treatment in the 21st century, a UK retrospective cohort study indicated that survival rates of patients 5 years post-diagnosis remain at ∼50% (Jones et al., 2019). To address this, researchers have been exploring innovative approaches to improve our mechanistic understanding and devise new treatment options to turn heart failure into heart success for patients.

In recent years, Disease Models & Mechanisms (DMM) has published a growing number of articles that propel preclinical and translational research in heart failure forward. Our archive of Open Access primary Research and Resource articles, as well as comprehensive Reviews, showcases the important progress this field has made in developing tools and preclinical models to improve our understanding of the mechanisms of heart failure and develop new therapeutic strategies.

Model systems that recapitulate the physiology and pathology of the human heart, from *in vitro* single-cell platforms and 3D cardiac organoids to small and large animals and even organ-level computational models, are instrumental in driving this progress. For example, a recent Resource article describes a robust platform for high-quality recordings of *in vivo* adult zebrafish electrocardiograms that will improve our ability to investigate mechanisms and therapies for a wide spectrum of human cardiac disorders in zebrafish models (Duong et al., 2021). The extensive array of new laboratory model systems can be used to delineate cardiogenesis. Improved understanding of cardiac development is essential to tackle congenital heart disease (CHD), the most common birth defect worldwide, which can often progress to heart failure. As recently reviewed by Rufaihah et al. (2021), these systems can improve mechanistic understanding to facilitate translatability to the clinic and development of novel CHD therapeutics. For example, Peterson et al. (2020) used a mouse model of congenital bicuspid aortic valve (BAV) to unpick the mechanisms underlying the associated aortopathy. They found that disrupted endothelial-mediated nitric oxide signalling inhibits elastic fibre formation, which causes both BAV and aortic dilation. Furthermore, a recent Resource article presented the first *in utero* surgically induced mouse model of isolated hypoplastic left heart syndrome. The mice survived to term and fully recapitulated the patient phenotype (Rahman et al., 2021). This model will allow researchers to interrogate the mechanisms and therapeutic targets of cardiac hypoplasia. CHD encompasses various disorders with diverse genetic causation; therefore, advanced tools are essential for untangling the complex pathological mechanisms and successful clinical translation.

Cardiomyopathies comprise a group of diseases that affect all ages and are characterised by weakening of the heart muscle, often resulting in arrhythmia, cardiac remodelling, heart failure and sudden cardiac death. Patient studies have revealed several disease-associated genes; however, animal models are key to furthering our mechanistic understanding of these mutations in cardiomyopathies. Recent studies have developed *Drosophila* models to recapitulate different forms of cardiomyopathies by mutating homologues of *ELAC2*, a gene important for transfer RNA (tRNA) maturation (Migunova et al., 2021), and *MYOM2*, which encodes a major component of the sarcomere (Auxerre-Plantié et al., 2020). Similarly, *Drosophila* were used to reveal a role for sphingosine Δ4 desaturase activity in lipotoxic cardiomyopathy (Walls et al., 2020). These studies revealed novel mechanistic insights into the genetics of cardiomyopathy and highlighted how tractable model animals, like *Drosophila*, can help delineate the heterogeneity of this group of disorders.

Cardiomyopathy is also a primary cause of early mortality in Duchenne muscular dystrophy (DMD), a disease characterised by progressive muscle wasting that is featured in our subject collection ‘Neuromuscular Disease Models’ (van Putten et al., 2020a). Animal models that fully recapitulate DMD-associated cardiomyopathy address the paucity of our understanding of the mechanism of this clinical manifestation and how it can be targeted therapeutically. A recent Review assessed the plethora of mouse models for muscular dystrophies, including DMD, but not all displayed impaired cardiac function (van Putten et al., 2020b). However, subsequent studies have described and fully characterised cardiac abnormalities in novel mouse (Wong et al., 2020), rat (Szabó et al., 2021) and porcine (Stirm et al., 2021) models of DMD. *In vitro* approaches are also valuable, as demonstrated by a recent study showing that exosomes secreted by DMD cardiomyocytes differentiated from human stem cells exacerbated the vulnerability of DMD cardiomyocytes to stress (Gartz et al., 2020). This expanding toolkit for exploring DMD-associated cardiac impairment will potentiate research in this field and propel clinical progress.

Many therapeutic avenues for heart failure are being explored. A preventative but also therapeutic option for heart failure is lifestyle alteration, including exercise. Murphy et al. (2021) demonstrated the power of zebrafish in modelling age-associated impairments in cardiomyocyte turnover and cardiac function, which could be ameliorated with increased activity throughout adulthood. Although this study suggests that a comparatively simple intervention can have clinical benefit, more effective drugs are also required. The number of new cardiovascular drugs entering clinical trials from 1990 to 2012 has declined (Hwang et al., 2016). Therefore, novel actionable pathophysiologic pathways in heart failure need to be uncovered. Interrogating mechanisms of regeneration of the myocardium is a major goal of the cardiovascular research community. Importantly, some species have a propensity for cardiac regeneration, and understanding the mechanisms of this phenomenon could enable the development of therapeutics for patients, as reviewed by Price et al. (2019). There have been impressive advances in adult pig and mouse preclinical models of cardiac regeneration (Price et al., 2019). However, deploying Cre recombinase to express pro-regenerative genetic programmes in transgenic mice can cause off-target DNA damage in cardiomyocytes (Wang et al., 2020). This raises important considerations for genetically engineered models to study cardiac regeneration; addressing these will ultimately help to improve continuing research in this area.

Cardiovascular research is an expansive and evolving area. With limitations in existing drug efficacy and transplantation availability, there is an urgent need to explore more innovative therapeutic options. In our upcoming Special Issue (Box 1), we aim to curate a collection of breakthrough research focused on the dysregulation of pathways, disease progression and approaches to treat and modify the course of heart failure using *in vitro* and *in vivo* model systems. This Open Access collection will advance our understanding of the mechanisms that orchestrate the varying forms of heart failure and potentiate therapeutic discovery.

**Box 1. Call for papers**

To continue our commitment to this important field of biomedical research, we are launching a Special Issue and ongoing subject collection, ‘Moving Heart Failure to Heart Success: Mechanisms, Regeneration & Therapy’. This issue will be coordinated by guest editors Jeroen Bakkers (Hubrecht Institute, The Netherlands), Milena Bellin (Department of Biology, University of Padova, Italy, and Leiden University Medical Center, The Netherlands) and Ravi Karra (Duke University School of Medicine, USA). The Special Issue will focus on the mechanisms and therapies for congenital heart disease and heart failure. Although heart failure is frequently related to cardiac ischaemia or high blood pressure, which have extensive coverage, this Special Issue will focus on heart failure associated with congenital heart disease, cardiomyopathy, arrhythmias, heart valve abnormalities and inflammatory disorders. We also welcome advances in the development of new model systems, resources, datasets and technologies for studying heart pathophysiology, repair and regeneration. We invite you to submit your breakthrough research to this Special Issue by Monday 1 August 2022.

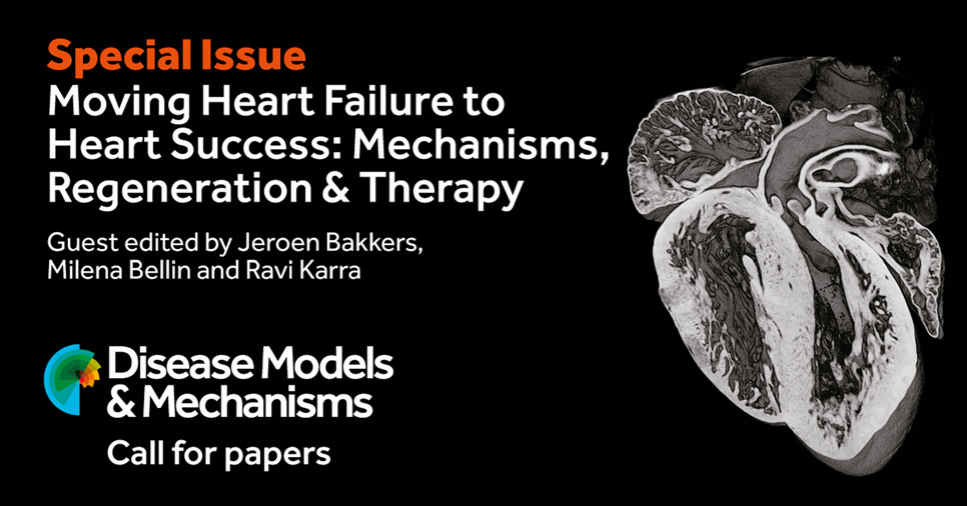

Your submissions will be handled by expert academic Editors and will receive high visibility and impact. We allow format-free submission, and our continuous publication model means that DMM will publish your article as soon as it is accepted. DMM is an Open Access journal, and we are included in a growing number of Read & Publish agreements with institutional libraries and library consortia, allowing corresponding authors at these institutes to publish an uncapped number of Open Access Research articles without paying an article processing charge. After the publication of this Special Issue in spring 2023, we will launch an ongoing collection to continue our commitment to this area of research.
